# Looking at Flavonoid Biodiversity in Horticultural Crops: A Colored Mine with Nutritional Benefits

**DOI:** 10.3390/plants7040098

**Published:** 2018-11-07

**Authors:** Aurelia Scarano, Marcello Chieppa, Angelo Santino

**Affiliations:** 1 ISPA-CNR, Institute of Science of Food Production, C.N.R. Unit of Lecce, 73100 Lecce, Italy; aurelia.scarano@ispa.cnr.it; 2 National Institute of Gastroenterology ‘S. De Bellis’, Institute of Research, 70013 Castellana Grotte (Ba), Italy; mchieppa@yahoo.it

**Keywords:** biodiversity, biofortification, flavonoids, horticultural crops, nutritional quality

## Abstract

Flavonoids represent a wide group of plant secondary metabolites implicated in many physiological roles, from the attraction of pollinators to the protection against biotic or abiotic stresses. Flavonoids are synthetized in a number of horticultural crops that are important components of our daily diet. In the last decades, the consumption of vegetables rich in antioxidants has been strongly promoted from the perspective of prevention/protection against chronic diseases. Therefore, due to their nutritional importance, several attempts have been made to enhance flavonoid levels in species of agronomic interest. In this review, we focus on the flavonoid biodiversity among the major horticultural species, which is responsible of differences among closely related species and influences the qualitative/quantitative composition. We also review the role of flavonoids in the nutritional quality of plant products, contributing to their organoleptic and nutritional properties, and the main strategies of biofortification to increase their content.

## 1. Introduction

To date, more than 6000 different flavonoids have been reported among the species of the plant kingdom. Based on their chemical structure and modification, flavonoids have been classified into many subgroups, as chalcones, flavones, isoflavones, flavonols, anthocyanins, proanthocyanidins, flavanols, and aurones [1,2]. All these compounds are synthesized via the phenylpropanoid biosynthetic pathway starting from the common precursor, phenylalanine. The phenylpropanoid pathway can be divided in an early branch of biosynthesis, driven by enzymes responsible for the production of all the main precursors, and late branches of biosynthesis, which generate the downstream flow of flavonoids. The downstream and the lateral biosynthetic branches are important contributors of the great chemical diversity of these metabolites. A number of different enzymes, i.e., glycosyl/methyl transferases, isomerases, reductases, hydroxylases, and dioxygenases, are responsible of the main decorations of the final chemical structures of flavonoids. Despite some genetic variance in these reactions, the main skeleton of the flavonoid biosynthesis pathway is widely conserved in the plant kingdom. Each species synthesizes specific flavonoid subgroups, which constitutes a chemical signature expressing the biodiversity of these phytochemicals and the specialization acquired by each species during evolution.

The reasons explaining why flavonoids are strongly conserved along the plant kingdom lie mainly on their physiological functions. Their role as developmental regulators of auxin transport and catabolism and the protection against UV radiation are considered the most ancestral functions of these phytochemicals [3]. Other functions such as the attraction of pollinators, the interaction with the rhizosphere, and the protection against phytopathogens/predators have probably evolved later. Flavonoids and anthocyanins, often acting as co-pigments, are also main contributors to the beautiful colors of flowers and fruits and are important UV-B protectors in terms of their absorbance and antioxidant activities, reducing the possible consequences/damages triggered by ROS species in plant tissues. In fact, the expression of genes belonging to early flavonoid biosynthesis is often used as a marker of the UV-B photoreceptor pathway and acclimation response [4].

The chemical biodiversity of flavonoids reflects the metabolic plasticity of land plants and, as for other secondary metabolites, their adaptation to local conditions. The occurrence of flavonoids shows variation among different plant species, the genetic background, the geographical area, the developmental stage of the plant, and external factors, such as abiotic or biotic stresses. Human domestication has been another factor affecting the metabolic compositions in current horticultural crops and consequently their nutritional value.

## 2. Natural Flavonoids Variation in Horticultural Species

**Figure 1:**
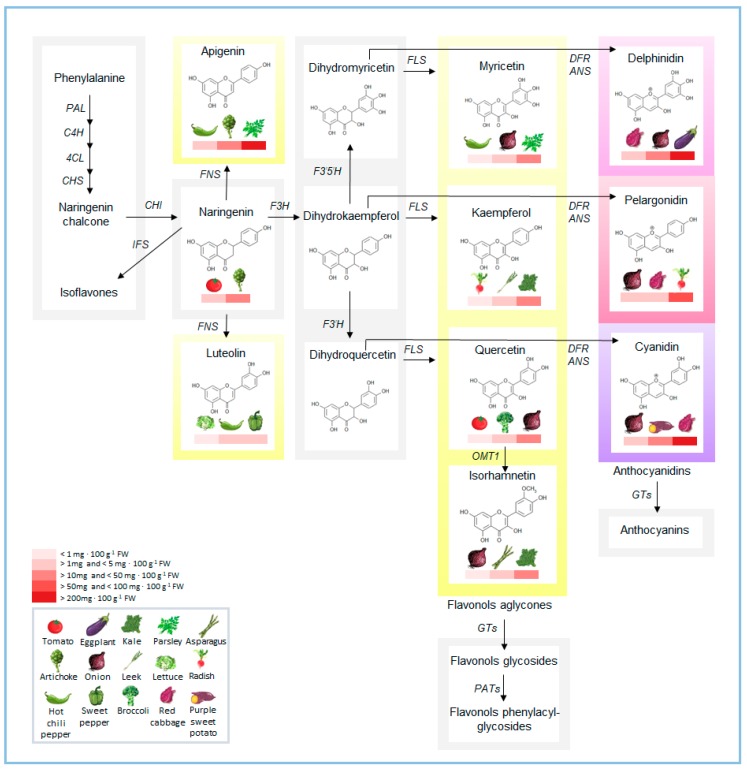
Schematic illustration of the flavonoid biosynthetic pathway, with the main flavonoids in representative horticultural species. Amounts of flavonoids (from USDA database; https://www.ars.usda.gov/nutrientdata [15]) are indicated as a red heatmap ranging from the species with lower to those with higher content. PAL, Phenylalanine ammonia lyase; C4H, cinnamic acid 4-hydroxylase; 4CL, 4-coumarate:coenzyme A ligase; CHS, chalcone synthase; CHI, chalcone isomerase; IFS, isoflavone synthase; FNS, flavone synthase; F3H, flavanone-3-hydroxylase; F3’H, flavanone-3’-hydroxylase; F3’5’H, flavanone-3’5’-hydroxylase; FLS, flavonol synthase; DFR, dihydroflavonol reductase; ANS, anthocyanidin synthase; OMT1, *O*-methyltransferase 1; GTs, glycosyltransferases; PATs, phenylacyl-transferases.

Table 1 and Figure 1 report the main flavonoids and their amounts detected in common horticultural crops. The qualitative/quantitative traits of flavonoid composition reflect species-specific steps of glycosylation, methylation, or other reactions which add other groups to the main backbone. The addition of glucose residues, or other sugars like arabinose or rhamnose, is a common modification step of flavonoids, together with acylation or even phenyl-acylation, producing very diverse and sophisticated compounds. Glycosylation of flavonoids is mediated by UDP-sugar-dependent glycosyltransferases (UGTs) that are active in the cytosol [5]. Glycosylation increases the solubility of flavonoids, important feature for storage inside vacuole or their transport to cell wall [6]. The great part of information on these processes derives from *Arabidopsis*, in which the flavonoid glycosylation has been well studied, and many glycosyltransferases have been identified and functionally characterized (i.e., flavonol 3-*O*-rhamnosyltransferase (F3RhaT, UGT78D1), flavonol 7-*O*-glucosyltransferase (F7GlcT, UGT73C6), a flavonoid 3-*O*-glucosyltransferase (F3GlcT, UGT78D2), flavonol 7-*O*-rhamnosyltransferase (F7RhaT, UGT89C1), flavonol 3-*O*-arabinosyltransferase (F3AraT, UGT78D3), flavonoid 3-*O*-glucoside 2″-*O*-glucosyltransferase (Fd3GGlcT, UGT79B6); 6 and references therein). Further information is available from purple potato and sweet potato in which UDP-glucose: flavonoid-3-*O*-glucosyltransferase have been as well characterized [7,8].

Acylation is another frequent modification occurring on flavonoids and anthocyanins. Cyanidin or pelargonidin glycosides have been found acylated or double acylated with *p*-coumaroyl, sinapoyl, caffeoyl, feruloyl, or malonyl functionalities in broccoli sprouts [9], red radish [10], red cabbage [11] or purple sweet potato [12]. More than 20 different non-acylated flavonol glycosides and acylated mono-, di-, tri-, and tetra-glycosides of quercetin, kaempferol, and isorhamnetin have been found in *Brassica rapa*. Interestingly, isorhamnetin derivatives are abundant in *B. rapa* group, but are usually absent in *B. oleracea* [11], thus confirming the strict specificity of lateral chain decorations among closely related species.

Several enzymes involved in anthocyanin- and flavonoid modifications, namely anthocyanin-3-*O*-glucosyltransferase (SlA3GlcT), anthocyanin-3-*O*-glucoside-6″-*O*-rhamnosyltransferase (SlA3Glc6″RhaT), anthocyanin-5-*O*-glucosyltransferase (SlA5GlcT), anthocyanin-3-*O*-rutinoside-4″-*O*-phenyltransferase, and flavonoid-3-*O*-rutinoside-4″-*O*-phenylacyltransferase (SlFd3Glc6″Rha4″PAT) have been identified in purple tomato [13]. This class of phenylacyl-transferases are also well conserved in *Solanaceous* species [13]. Some bibliographic sources suggest the presence of twenty-nine putative flavonol-phenylacylglycosides in *Brassicaceae*, even though the flavonol-phenylacyl glycosides found and annotated to date are only 2″- or 4″-*O*-phenylacylated-flavonols [14]. However, phenylacyl-transferases are not commonly shared in flavonol biosynthesis pathway, indicating a more recent adaptation and a more selective restriction in their occurrence in some species [14]. In *Arabidopsis*, the phenylacylated flavonols have been related to the response of UV-B light tolerance [14], indicating their specialization to counteract this abiotic stress.

## 3. Flavonoid Pathway in Horticultural Species

The molecular bases underlying the flavonoid biodiversity consist of a different activation of the genes along the pathway. Most of the key structural enzymes of the central flavonoid metabolism are encoded by single-copy genes, but others are encoded by multiple genes, as in the case of PAL, CHS, F3H, or FLS [39]. The expression of biosynthetic (structural) genes shows a large variation according to the species. The fine regulation of the biosynthetic genes is controlled in an organ- and tissue-specific manner by MYB, bHLH, and WD40 transcription factors (TFs), which act in an orchestrated ternary complex, indicated as MBW by the initials of each TF. MYBs can be necessary and sufficient regulators or can alternatively require the presence of bHLH and/or WD40 partners acting as co-regulators [40]. Another type of regulation is mediated by a post-transcriptional control, since for example the inactivation of PAL can occur by its phosphorylation in association to the enzymatic turnover [41].

In *Brassicaceae*, such as *B. rapa* and *B. napus*, orthologs of the *Arabidopsis Transparent Testa* (*TT*)-genes have been identified. For example, *BrTT8* regulates proanthocyanidins accumulation in seed coat and the expression of the late flavonoid biosynthetic genes [42]. In *B. napus*, out of the eighteen genes involved in the flavonoid pathway, nine of these are active in the early stages of seed development, and two as late structural biosynthetic genes. Interestingly, *BnTTG2* and *BnTT16* regulatory genes are putatively involved in seed color and orchestrate the expression of structural genes [43].

In onion, DNA sequences of several genes of the flavonoid pathway and their assignment to specific chromosomes have been reported [44]. Different loci have been involved in bulb color, as in the case of the *L* locus, corresponding to an anthocyanidin synthase gene, or the *R* locus, corresponding to a dihydroflavonol 4-reductase gene [45]. Furthermore, red onion AcMYB1 positively regulates anthocyanin biosynthesis pathway [46]. In a gold-colored onion line, the levels of transcripts of all of flavonoid biosynthetic genes are similar to those observed in red onions [47]. However, in this gold line, a premature stop codon in the coding region of the *CHI* gene is responsible of the inactivation of the *CHI* gene, thus inducing a block in the flavonoid biosynthesis pathway and the accumulation of chalcone derivatives, conferring the bright yellow color [47]. However, this is not an isolated case of CHI block, since a similar bottleneck in the flavonoid pathway has been also reported from tomato [48]. In this species, the introduction of heterologous CHI by transgenic approach resulted in increased levels of quercetin glycosides located downstream the CHI catalyzed step [49].

While in most horticultural varieties the activation of flavonoid and anthocyanin biosynthetic genes have been mainly observed in response to the light [50], in purple-colored carrots the reasons of this activation are still unclear. The purple or purple-orange carrots have their origins in the oriental countries and were commonly consumed before the domestication and introduction of the orange carrots occurred in the 17th century in Europe, probably in Netherlands [51]. However, some varieties and local landraces still exist [52,53], and in some cases the flavonoid and anthocyanin pathway has been investigated. Yildiz et al. (2013) [54] reported that five anthocyanins biosynthesis genes and three anthocyanin transcription factors map in a population segregating for the *P1* locus that influence the purple root color. Yet, either the structural biosynthetic genes [55] and TF [56] were found highly expressed in the purple carrots compared to the yellow or orange ones. One of these, the *DcMYB6* gene, shares high identity with anthocyanin-regulating MYBs from other species [56], but it remains unclear if DcMYB6 requires bHLH cofactors or its activation is sufficient to trigger the expression of anthocyanin genes in below ground tissues.

In red-purple spinach, the expression patterns of the *SoPAL*, *SoUFGT3*, *SoUFGT4*, and regulatory MYB, bHLH, and WD40 genes have been reported, thus confirming their important role in anthocyanin biosynthesis in this species [57]. In globe artichoke, the role of the regulatory gene *CcMYB12* was investigated in heterologous models, where it resulted in a positive regulation on the flavonol biosynthesis [58].

The accumulation of anthocyanins is one of the most studied pathways in potato, since pigmented potato cultivars are considered a good source of these phytochemicals at levels similar to blueberries, blackberries, cranberries, and grape [59]. Similarly to other species, potato shows the presence of several genetic loci specifically controlling anthocyanin biosynthesis [59]. Key regulators of the phenylpropanoid and anthocyanin pathway are StAN1, StAN2, StMYBA1, and StMYB113, but a crucial role is also played by bHLH co-factors, since StAN1 and StAN2 interact with StbHLH1 and StJAF13 in different organs, i.e., leaf and tuber [59,60]. Among other transcription factors encoding genes affecting the anthocyanin accumulation in potato, StAN11, a WD40-repeat gene, has been proposed as a regulator of the pathway by controlling the expression of DFR [61]. Furthermore, the ScAN2 MYB transcription factor has been found induced by low temperatures in the potato cold-tolerant species *Solanum commersonii*. Interestingly, ScAN2 is a paralog of ScAN1, even though ScAN2 is less able to induce anthocyanin than ScAN1, having in turn the ability to induce hydroxycinnamic acids [62]. 

Flavonoid biosynthesis and its regulation have been particularly studied in tomato over the years. In the flesh of this fruit, all flavonoid genes are scarcely expressed and flavonoids are accumulated in very low amounts. Conversely, the peel accumulates naringenin chalcone and rutin as major flavonoids detected during the fruit ripening process [1]. In this tissue, CHS, responsible of naringenin chalcone production, is highly expressed but not CHI, which is required to convert naringenin chalcone into naringenin. The low expression of CHI suggests that it is a rate-limiting step for flavonoid production in tomato [1]. The orthologue of the *Arabidopsis MYB12* gene [40,63] has been identified and characterized in tomato (SlMYB12) [64]. *SlMYB12* expression increases markedly during the green and green-yellow fruit developmental stages and it is implicated in caffeoyl quinic acids (CQAs) and flavonol biosynthesis [64]. Recent insights using a metabolomic and transcriptomic integrated approaches revealed implications of SlMYB12 in a more extended metabolic network (for example, some influences on polyamine biosynthesis pathway) [65]. In other studies, members of MBW complex have been identified as regulators of anthocyanin biosynthesis, such as SlANT1 and SlAN2 (MYB TFs) [66,67] both mapping to tomato chromosome 10. Recently, the WD40 TF named SlAN11 has been characterized and proposed to promote plant anthocyanin and seed proanthocyanidin (PA) contents [68].

## 4. Environmental Factors Affecting Flavonoid Composition in Horticultural Crops

Beside genomic factors, environmental conditions and agronomic practices also affect flavonoid levels in horticultural species. Flavonoid content shows deep variations during different stages of fruit ripening, but light and temperature can also greatly influence their levels. UV-B exposition can trigger the anthocyanin biosynthesis, whereas variations in the photoperiod, light intensity, and wavelength can stimulate differently the flavonoid pathway [69]. In fact, most of the structural and regulatory genes of this pathway are light-responsive [70]. Positive effects following light exposure have been found in tomato [71]. In broccoli and kale, flavonoids concentration increases with high photosynthetic active radiation (PAR) [72,73] and qualitative differences in flavonol content have been reported after UV-B radiation [74]. An improvement in the flavonoid content after UV-B irradiation has been also described in sweet basil [75]. Temperature can influence the rate of biochemical reactions catalyzed by different enzymes and affect phytochemical accumulation in plants [76]. Furthermore, during fruit development, variation in temperatures can affect photosynthesis, respiration, and secondary metabolism. Qualitative changes in the flavonol profile have been found after low temperature treatments between 0.3 and 9.6 °C in broccoli and kale [77].

Irrigation and nutrition are cultural practices that can further affect the flavonoid content in vegetables. Water stress has been reported inducing the transcription of genes encoding PAL, C4H, 4CL, CHI, and F3H [78]. Deficit of irrigation in three leafy lettuce varieties induced an increase of flavonols i.e., kaempferol, myricetin, and quercetin [79]. Water stressed lettuce plants have also displayed increased levels of total phenolic content and antioxidant capacity [80].

Several studies related the flavonoid content in horticultural crops to the presence of nitrogen or organic inputs in the soil [81]. Interestingly, organic fertilizers functioned as a sink from which the flux of primary metabolites feeds secondary metabolism. A decrease in flavonoids has been mainly correlated to nitrogen supply [81]. A possible explanation resides in the carbon/nutrient balance (CNB) hypothesis, according to which, low nitrogen availability induces an increase in carbon availability and thus carbon-based secondary metabolites [70]. In red basil [82] and broccoli [83], a decrease in flavonoid content has been observed following nitrogen fertilization. In another study, tomato fruits from plants grown with low nitrogen supply showed a higher phenolic content [84]. In a field experiment on broccoli cultivars, high flavonoids levels have been detected in response to soil organic fertilization. However, another study, in which organic treatment and winter cover crop was experimented, did not result in any significant increase in flavonoid content [85]. These data suggest that agronomic practices, i.e., organic or conventional management, growing location (growth in plots or fields, in greenhouse or open fields) and environmental conditions (light, temperature) have to be carefully applied and evaluated for each plant species in the perspective of increasing flavonoid content [81].

## 5. Effects of Processing on Flavonoids Content

Stems, leaves, roots, inflorescences, or fruits of horticultural crops are generally consumed as fresh foods or often, variously processed in industrial or homemade preparations. Processing of fresh food products can generally modify organoleptic and nutritional properties, since many volatile compounds or phytochemicals like macro or micronutrients (carbohydrates, amino acids, minerals, vitamins, phenols, etc.) can be transformed or destroyed. Crushing, pressing, and temperature-dependent treatments can deeply impact on the flavonoid content [86], with consequences on their bioactivity. These processes generally disrupt in mechanic way plant tissues and subsequently cell wall, thus favoring the solubilization of phytochemicals. Mechanical disruption can break the structure itself of phytonutrients and in addition, it can release enzymes that can promote the degradation of biocompounds. For example, flavonoids can be oxygenated and degraded by several enzymes (polyphenol oxidases, peroxydases, glycosidases, esterases) that are released when plant cells are broken down. Glycosidase and esterase enzymes catalyze flavonoid degradation during hydrolysis reactions. Oxidation reactions caused by polyphenol oxidases and peroxidases tend to blacker pigments, thereby having negative effects on quality of fresh vegetables and fruits [87].

In asparagus spears, domestic preparations like peeling significantly reduced flavonoid content, after storage at 2 °C [88]. Similarly, cutting has been showed to reduce significantly the content of rutin in this species [89], but this practice seems to not affect quercetin 3,4’-glucoside and quercetin 4’-glucoside content, and the overall antioxidant capacity of onion [89].

High temperatures were also reported as one of the main treatment responsible of the reduction of flavonoids and anthocyanins in processed fruits and vegetables (as in the case of flavonols of asparagus and onions; [89]), and phenolic compounds and flavonoids (particularly kaempferol and quercetin, in carrots, courgettes, and broccoli; [90]). In tomato, a 80%, 65%, and 30% reduction in quercetin levels were reported after boiling, microwave cooking, and frying, respectively [91].

This large literature body clearly points that processing strongly modifies the phytochemical composition of fruits and vegetables, with a general decrease in their nutritional quality. However, beside the fact that cooking makes foods more digestible, high temperature treatments are usually employed to destroy microorganisms and sanitize food products. Emerging alternatives to these invasive treatments i.e., mild non-thermal treatments, as in the case of high-pressure and plasma treatments have been proposed [92], providing the possibility to use these technologies to preserve food from pathogen contamination and unwanted enzymatic activities and at the same time to potentially extend the shelf life and nutritional value of food crops.

## 6. Flavonoids Biofortification

Biofortification consists of a number of strategies aimed at increasing the content of compounds considered beneficial for human health. Such an increase can be reached by application of specific fertilizers for plant nutrition, but also using breeding or metabolic engineering approaches by which it is possible to increase the synthesis of phytochemicals or even induce the production of new compounds without any intervention during the pre/post-harvest steps [93]. Breeding or metabolic engineering programs in the context of flavonoid biofortification has been usually used to enrich crop species that naturally are unable to accumulate optimal amounts of specific bio-compounds. As for other phytochemicals, the enrichment of flavonoids by breeding depends on the availability of genetic resources and variability. Genetic screening of wild cultivars and landraces is helpful to identify specific traits which can be later introgressed in domesticated varieties [94]. Potato breeding strategies for anthocyanin enhancement have been proposed using some South American cultivars, as interesting sources of flavonoids [62]. Another good example of anthocyanin improvement refers to tomato. In this species, the dominant *Aft* (Anthocyanin fruit) gene has been introgressed in a domesticated cultivar by crossing with *S. chilense* and the recessive *atv* (atroviolacea) gene by interspecific cross with *S. cheesmaniae*. The resulting tomato plants were double mutants, carrying both Aft and atv alleles (Aft/Aft atv/atv), and accumulated anthocyanins in the fruit skin until ripening, strongly stimulated by light and cold [95]. Another interesting example of breeding for anthocyanin enrichment has been realized using a white Chinese cabbage as female parent and zicatai (*Brassica rapa* L. ssp. *chinensis* var. *purpurea*) as male parent, resulting in a purple heading Chinese cabbage line rich in anthocyanins [96,97].

Wild garlic germplasm has been also used as a viable option for breeding strategies to increase the flavonoid content in this species. *Allium ursinum* (wild garlic) and *A. victorialis* contain genetic traits for novel flavonoids, which could be introgressed in *A. sativum*. So far, garlic breeding has been limited to clonal selection of wild varieties or natural mutants, but a recently developed routine seed production will be useful for introgression of genes from wild relatives [98].

**Figure 2:**
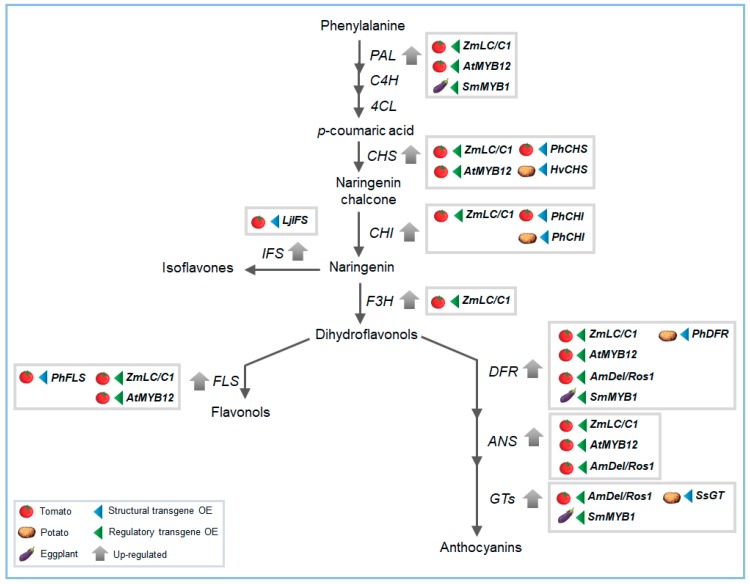
Flavonoid biofortification by metabolic engineering strategies through the use of structural (blue arrow on the right of the boxes) or regulatory genes’ (green arrow on the left of the boxes) over-expression (OE). The scheme reports the main genes targets studied for flavonoids improvement in horticultural species.

Metabolic engineering can be also a useful tool for flavonoids enrichment. So far, two strategies that make use of structural or regulatory genes have been developed, sometimes used in combination (Figure 2). The first one consists of the introduction of novel branches, mediated by transgenic insertion of new genes not present in the genome, or alternatively the selective inactivation of genes involved in metabolic fluxes [1,63]. The second strategy consists of over-expression of genes encoding transcription factors that modulate different steps of the pathway [1]. For example, a single or simultaneous over-expression of *CHS*, *CHI*, and *DFR* structural genes resulted in a significant increase in flavonoid and anthocyanins in potato, accompanied by a decrease of starch and glucose levels [99]. A potato UDP-glucose:flavonoid-3-*O*-glucosyltransferase (*3GT*) has been over-expressed resulting in increased anthocyanins content in tuber skin [100]. Another study reported high amounts of anthocyanins, kaempferol, chlorogenic acid, sinapic acid, and proanthocyanins in transgenic potato plants over-expressing *DFR* and *UGT* [101].

In tomato, one of the first attempts to improve flavonoid production made use of structural genes limiting the flavonoid biosynthesis. The over-expression of *Petunia CHI* gene, as an example, resulted in increase of the flavonol content in the peel [49,102]. Another strategy to improve flavonoids content consisted of the use of regulatory genes. For example, ectopic over-expression of the maize leaf color (*LC*) and colorless *C1* regulatory genes led to the accumulation of anthocyanins in leaves and other flavonoids like kaempferol glycosides in the fruit of tomato [103]. An increase in flavonoid levels has been also reached by suppressing the *DET1* (De-etiolated) regulatory gene combining a RNAi approach with the use of fruit-specific promoters [104]. Zhang et al. (2016) [105] showed that the introduction of SmMYB1 into a non-anthocyanin-producing eggplant cultivar, induced high accumulation of anthocyanin in plant tissues and highly methylated anthocyanins mainly in fruit flesh, with a higher tolerance to freezing stress observed in transgenic lines. High accumulation of anthocyanins in both flesh and peel of tomato fruit has been reached by using the snapdragon transcription factors Delila and Rosea [106]. Furthermore, the over-expression of the AtMYB12 in tomato was successful to reach high levels of CQAs and flavonols in the whole fruit [64]. These examples clearly indicate the great potential of the transcription factors as valuable biotechnological tools for an effective enhancement of nutritionally important phytochemicals.

A combination of different TFs was also employed to obtain high accumulation of different classes of flavonoids, as it has been reported for the Indigo tomato line, obtained by crossing the lines over-expressing AtMYB12 and Del/Ros [63]. This work case also demonstrated the “pushing” function that the AtMYB12 transcription factor exerts on the whole flavonoid metabolic flux. In fact, the combined over-expression of the *AtMYB12* gene and the *IFS* (isoflavone synthase) gene resulted in the accumulation of high levels of flavonols and isoflavones in the whole fruits. The crossing of transgenic lines has been also described in another study, in which tomato lines over-expressing the onion *CHI* gene and the *Arabidopsis PAP1* (Production of Anthocyanin Pigment 1) gene, upregulating the early biosynthetic genes, were crossed to obtain CHI x PAP1 lines. The resulting tomato line accumulated higher levels of rutin and anthocyanins in the fruit skin compared to wild type tomatoes [107]. If in most of the cases, flavonoid biofortification has been realized by classic transgenic approaches, emerging genome editing technologies are revealing their great potential in the improvement of plant food quality. CRISPR/Cas9-mediated genome editing is one of the most promising technologies to reach this scope. Despite many efforts, studies are still needed, and some data indicate that biofortification is possible through this approach [108,109], even though a molecular editing plan, plant regeneration, and accurate screening methods have to be carefully addressed for each plant species [109].

## 7. Flavonoids in Plant Food Quality and Nutritional Aspects

Flavonoids contribute to the quality of horticultural crops, contributing either to their organoleptic or nutritional quality. Color is one of the sensory parameters highly determining the degree of acceptability of plant food products. Flavonoids, and especially anthocyanins, are among the most popular natural pigments conferring the color to fruits and vegetables, together with chlorophylls (green), carotenoids (yellow, orange and red), and betalains (red and purple) [110]. Flavonoids like flavanols (i.e., catechins and epicatechins), flavonols (i.e., rutin, quercetin), flavanones (i.e., hesperetin, naringenin), chalcones, and flavones (i.e., luteolin) usually contribute to the range of ivory/bright yellow colors in plant foods.

Flavonoids like isoflavones or proanthocyanidins play also an important role in the sensation of bitterness and astringency. The perception of bitterness has been linked to structural signatures, such as the presence of aromatic rings or carboxylic acid groups [111]. Sensation of astringency has been reported to be due to the precipitation of salivary proteins following their interaction with polymeric proanthocyanidins [112], a perception that makes them unpalatable and can be considered a defense mechanism of plants towards herbivorous animals [113].

Considering food quality, anthocyanins, for example, act as antioxidant compounds contrasting ROS activities, thus extending the shelf-life of fruits [114,115]. Furthermore, flavonoids display antibacterial properties against a wide range of pathogenic microorganisms. Several mechanisms have been proposed, ranging from the inhibition of nucleic acid synthesis and biofilm formation to the alteration of membrane permeability [116]. 

Flavonoids contribute significantly to the nutritional properties of plant food and their accessibility to human gut is an important feature. In fact, the bio-efficacy of flavonoids is largely dependent on their bioavailability. For example, the physical and chemical composition of food matrices may delay or enhance flavonoids digestibility and absorption. Proteins, dietary fibers (hemicellulose, pectins), lipids, and other phytochemicals can also influence their bioavailability. For example, the carbohydrate composition of the food matrix has been reported to modulate flavonol absorption in the small intestine [117].

In the human body, flavonoids undergo several modifications catalyzed by enzymatic activities during absorption and digestion and, afterwards, they are substrates of biotransformation occurring by gut microbiota. Reactions of oxidation or hydrolysis to release aglycones, or the conjugation with O-glucuronides or methyl esters in the enterohepatic circle, have the function to increase the hydrophilicity and render flavonoids more transportable in the bloodstream, charged by plasmatic proteins, and render them more absorbable in the small intestine. Uptake of flavonoids at the intestinal barrier is generally mediated by passive diffusion or transporters located into the lipid bilayer of intestinal epithelial cells membrane [118,119]. Isoflavone aglycones are transported into enterocytes more efficiently than their glycosylated forms, due to their lipophilicity [119] and easier absorption [120]. Transport of quercetin 4′-β-glucoside across the apical membrane of enterocytes has been reported mediated by SGLT1 (sodium-dependent d-glucose cotransporter-1) [121]. (−)-epichatechin-3-gallate, a flavan-3-ol, is absorbed by the monocarboxylic acid transporter (MCT), as well as ferulic acid, *p*-coumaric acid, or caffeic acid [122]. Rutin and naringin are mainly absorbed from the distal part of the intestine after hydrolysis by colonic microflora intestinal enzymes and β-glucosidases [122].

In the large intestine, flavonoids can be further converted by gut microbiota in simple aromatic compounds. Even though flavonoids are biotransformed at the intestinal level, byproducts obtained from these reactions can still be active and exert antioxidant or anti-inflammatory properties [123]. Flavonoids are also known to modulate microbiota, inducing a remodelling of bacterial communities and genera in the microbiota environment, thus acting as prebiotics [124].

Although there are many studies reporting the beneficial activities of flavonoids and anthocyanins, the majority of them consider the administration of synthetic compounds (pure flavonoid standards), which does not fully reflect the effects of the whole food matrices of fruits and vegetables. From this point of view, it is important to elucidate the role of flavonoids on human health within their natural matrices and normal diet conditions. Focusing on this type of study, in vitro and in vivo assays have shown the following nutraceutical benefits:

(i) **Anti-cancer activity**. Among the major mechanisms studied to test the efficacy of flavonoids in the protection against different types of cancer, there are the induction of apoptosis in vitro or reduction of tumor incidence in vivo, and the inactivation of p53 protein, which plays an important role in cell cycle regulation, tumor suppression, and protection against oxidative damage. In a cell proliferation assay on human breast cancer cell lines, crude onion extracts showed a higher cytotoxic activity than purified quercetin and kaempferol [125]. Purple potatoes exerted positive effects against colon cancer stem cells in mice, with a suppression of tumor incidence [126]. In another study, a purple-fleshed sweet potato diet induced preventive benefits without toxicity in a colorectal cancer mouse model [127]. A significant increase of the average life span has been shown in *Trp53^−/−^* knockout mice fed with purple tomato-enriched diet, suggesting a protective effect against cancer progression [106].
(ii) **Hypoglycemic effects and antioxidant activities**. Some studies have investigated the positive hypoglycemic effects of onion skin extract on diabetes mellitus and spikes in postprandial blood glucose in animal models ([128] and references therein). Flavonoids from artichoke extracts lowered blood sugar in normal and obese rats [129], even if the mechanism of action has not been elucidated. The beneficial effects in lowering glucose can be linked to the ability of flavonoids to reduce vascular nitric oxide (NO) production, lipid peroxidation, and the pre-oxidant enzymes (superoxide dismutase, catalase, and glutathione peroxidase), all implicated in the onset of tissue oxidative stress, involved in several human diseases. This fact is particularly interesting considering that the state of oxidation/antioxidation imbalance generates complications in a number of human pathologies i.e., diabetes and obesity [130]. A study on endothelial cells showed that artichoke flavonoids upregulate the nitric-oxide synthase (eNOS) expression, an effect particularly important in regulating vascular function. In fact, eNOS balances vasodilatation and vasoconstriction mediated by nitric oxide (NO) and superoxide, respectively [130].
(iii) **Anti-bacterial activity**. In vitro studies suggest an antimicrobial activity of onion extract on pathogenic isolates of *Escherichia coli*, *Staphylococcus aureus*, *Streptococcus*, and *Streptococcus pneumonia* [131]. Garlic extracts are also well known for showing antimicrobial activities [132]. The antibacterial activity of flavonoids might be due to molecular interactions inducing inactivation of microbial proteins, like adhesins or membrane transport proteins, altering membrane permeability, and interfering with cell growth [124,133]. Different behavior between Gram-positive and Gram-negative bacteria has been reported after flavonoid exposure. This could be likely explained by differences in cell wall composition and can explain at least in part the prebiotic effects on the bacterial communities in human gut microbiota [123].
(iv) **Anti-inflammatory and immune-modulatory activities**. In vitro data indicate that flavonoids can modulate the inflammatory response, interfering in the major intracellular signaling pathways (i.e., NFkB, MAPKs) and decreasing the production of pro-inflammatory interleukins [134]. In a model of murine colonic epithelial cells, the administration of high-flavonols and high-anthocyanins tomato extracts reduced the production of IL-6 and TNFα interleukins, and the migration of primary leukocytes and dendritic cells, which are implicated in the immune responses. Furthermore, high-flavonols and high-anthocyanin tomato extracts inhibited the SAPK/JNK and p38MAPK inflammatory signaling pathways [135]. Administration of a tomato diet enriched in flavonoids and anthocyanins contributed to the reduction of inflammatory bowel diseases symptoms in a mouse model of ulcerative colitis [136]. Finally, red onion extracts have also shown immune-modulatory effects in an experimentally induced prostatic hyperplasia of rats [137].

## 8. Concluding Remarks

A growing body of evidence highlights the great versatility and plasticity of the flavonoid secondary metabolism among land plant species, focusing many efforts on the biodiversity and complexity of their chemical structures. Nevertheless, other studies are necessary to better elucidate flavonoid metabolism (for example, phytochemical composition, biosynthesis regulation, and genetic bases) in many less-studied horticultural crops. The chemical variety represents a mine of information either on the evolution/adaptation at specific (and often restrictive) environmental conditions, or the nutritional value of edible species. The biofortification strategies aimed at the improvement of these phytochemicals and thus the nutritional value of plant food products will offer the opportunity to develop new enriched plant foods, useful either in healthy or pathological conditions in the prevention or as pharmacological treatment adjuvant of important human pathologies. Further information from studies testing the effects of whole food matrices using more accurate animal models of important human pathologies will, therefore, be crucial to define the suitable amounts of specific phytochemicals in customized nutritional programs.

## Figures and Tables

**Table 1:** Flavonoid classes and content in horticultural crops. Types and amounts of flavonoid are reported either from the USDA database (USDA; https://www.ars.usda.gov/) [15] or from literature. USDA source refers specifically to flavonoid content evaluated by column chromatography or HPLC analyses and are expressed as mg/100 g FW of edible portion. The literature source refers to either HPLC or spectrophotometric quantifications, with the aim to provide further information on flavonoid amounts and the total flavonoid content in horticultural crops. TFC: total flavonoid content. NA: not available.

Family	Scientific Name/Common Name	Class of Flavonoids	Types and Content of Flavonoids (mg·100 g^−1^ FW, from USDA)	Types and Content of Flavonoids (Other Sources)
Brassicaceae	*Brassica oleracea* var. *acefala*/ Kale	Flavonols	Isorhamnetin 23.60 Kaempferol 46.80 Quercetin 22.58	Isorhamnetin 60 mg/Kg FW Kaempferol 24 mg/Kg FW Luteolin 25 mg/Kg FW [16]
	*Brassica oleracea* var. *capitata*/ Cabbage	Flavonols Flavones	Quercetin, 0.28 Kaempferol, 0.18 Luteolin, 0.10 Apigenin, 0.02	TFC 40 mg QE/g FW Kaempferol 31 mg/Kg FW Luteolin 33 mg/Kg FW [16,17]
	*Brassica oleracea* var. *capitata* f. *rubra*/ Red Cabbage	Flavonols Flavones Anthocyanins	Quercetin, 0.36 Myricetin, 0.20 Luteolin, 0.10 Apigenin, 0.06 Cyanidin, 209.83 Delphinidin, 0.10 Pelargonidin, 0.02	TFC 842 mg CE/Kg FW TFC 74 mg QE/g FW Cyanidin 40–750 mg/Kg FW [17,18,19]
	*Brassica rapa* var. *Pekinensis*/ Chinese cabbage	Flavonols	Kaempferol, 22.51	TFC 57 mg QE/g FW Kaempferol 33 mg/Kg FW Isorhamnetin 46 mg/Kg FW [16,17]
	*Brassica oleracea* var. *botrytis* Cauliflower	Flavonols Flavones	Quercetin, 0.54 Kaempferol, 0.36 Luteolin, 0.09 Apigenin, 0.03	TFC 267.21 mg CE/100 g DW Isorhamnetin 40 mg/Kg FW Kaempferol 7 mg/Kg FW Quercetin 2 mg/Kg FW Luteolin 13 mg/Kg FW Apigenin 5 mg/Kg FW [16,20]
	*Brassica rapa*/ Turnip	Flavonols	Kaempferol, 11.87 Quercetin, 0.73	Isorhamnetin glycosides 2–5 mg/Kg DW (stem/leaves) Isorhamnetin glycosides 6.5 mg/Kg DW (flower buds) [21]
	*Raphanus sativus*/ Radish	Flavonols Anthocyanins	Kaempferol, 0.86 Pelargonidin, 63.13	TFC 179 mg CE/Kg FW TFC 60–96 mg/g DW Pelargonidin glycosides 1.80 mg/g DW Kaempferol 4 mg/Kg FW [16,18,22]
	*Brassica oleracea* var. *italica*/ Broccoli	Flavonols Flavones	Kaempferol, 7.84 Quercetin, 3.26 Luteolin, 0.80 Myricetin, 0.06	Kaempferol 21 mg/Kg FW Quercetin 5 mg/Kg FW Quercetin 0.03–10.85 mg/100 g FW Kaempferol 0.24–13.20 mg/100 g FW [16,23]
Liliaceae	*Allium cepa*/ Onion	Flavonols Flavones	Quercetin, 20.30 Isorhamnetin, 5.01 Kaempferol, 0.65 Myricetin, 0.03	TFC 270–1187 mg/Kg FW TFC 170 mg CE/Kg FW (mostly quecetin, kaempferol and isorhamnetin derivatives) Kaemfperol 41 mg/Kg FW Quercetin 14 mg/Kg DW Apigenin 26 mg/Kg FW [16,18,24]
	*Allium cepa*/ Red onion	Flavonols Flavones Anthocyanins	Quercetin, 39.21 Isorhamnetin, 4.58 Myricetin, 2.16 Kaempferol, 0.70 Apigenin, 0.24 Luteolin, 0.16 Delphinidin, 4.28 Cyanidin, 3.19 Peonidin, 2.07 Pelargonidin, 0.02	TFC 415–1917 mg/Kg FW Total Anthocyanins 39–240 mg/Kg FW (mostly cyanidin glycosides) Kaempferol 48 mg/Kg FW Quercetin 10 mg/Kg FW Apigenin 34 mg/Kg FW [16,24]
	*Allium ampeloprasum*/ Leek	Flavonols Flavones	Kaempferol, 2.67 Myricetin, 0.22 Quercetin, 0.09	Quercetin 1.56 mg/g DW Rutin 1.04 mg/g DW Kaempferol 118 mg/Kg DW Quercetin 9 mg/KG FW Luteolin 33 mg/Kg FW [16,25]
	*Allium sativum*/ Garlic	Flavonols	Quercetin, 1.74 Myricetin, 1.61 Kaempferol, 0.26	TFC 100 mg/g aged extract; 47 mg/g fresh extract TFC 94 mg/g aged extract; 43 mg/g fresh extract [26]
	*Asparagus officinalis*/ Asparagus	Flavonols	Quercetin, 13.98 Ishoramnetin, 5.70 Kaempferol, 1.39	TFC 4.7 mg RE/g DW [27]
Apiaceae	*Daucus carota*/ Carrot	Flavonols Flavones	Kaempferol, 0.24 Quercetin, 0.21 Luteolin, 0.11	TFC 4.7–9.9 mg QE/100 g FW Isorhamnetin 41 mg/Kg FW Kaemfperol 5 mg/Kg FW Quercetin 9 mg/Kg FW Luteolin 22 mg/Kg FW [16,28]
	*Daucus carota* spp. *sativus* var. *atrorubens*/ Purple carrot	Anthocyanins	NA	Total anthocyanins 1.5–17.7 mg Cy3GE/100 g FW (mostly cyanidin, pelargonidin and peonidin galactosides) [29]
	*Apium graveolens*/ Celery	Flavonols Flavones	Quercetin, 0.39 Kaempferol, 0.22 Apigenin, 2.85 Luteolin, 1.05	Kaempferol 5 mg/Kg FW Apigenin 139 mg/Kg FW Luteolin 23 mg/Kg FW [16]
	*Petroselinum crispum*/ Parsley	Flavonols Flavones	Myricetin, 14.84 Kaempferol, 1.49 Quercetin, 0.28 Apigenin, 215.46 Luteolin, 1.09	TFC 14 mg QE/g DW Kaempferol 18 mg/Kg FW Quercetin 5 mg/Kg FW Isorhamnetin 11 mg/Kg FW Luteolin 14 mg/Kg FW Apigenin 4 mg/Kg FW [16,30]
	*Coriandrum sativum*/ Coriander	Flavonols	Quercetin, 52.90	Quercetin 19 mg/Kg FW Isorhamnetin 13 mg/Kg FW [16]
Asteraceae	*Lactuca sativa*/ Lettuce	Flavonols Flavones	Quercetin, 4.16 Myricetin, 0.07 Apigenin, 0.13 Luteolin, 0.26	Quercetin 32.2 mg/Kg FW Isorhamnetin 12 mg/Kg FW Luteolin 2 mg/Kg FW [16]
	*Cynara cardunculus*/ Cardoon		NA	TFC 7 mg CE/g DW (stem) [31]
	*Cynara scolymus*/ Artichoke	FlavanonesFlavones	Naringenin, 12.50 Apigenin, 7.48 Luteolin, 2.30	TFC 13 mg CE/g DW (stem) [31]
	*Chicorium endivia*/ Endive	Flavonols Flavones	Kaempferol, 10.10	Quercetin 30 mg/Kg FW Isorhamnetin 7 mg/Kg FW Luteolin 9 mg/Kg FW [16]
	*Chicorium intybus*/ Chicory	Flavonols Flavones	Quercetin, 6.49 Kaempferol, 2.45 Luteolin, 2.08 Apigenin, 0.77	TFC 20–120 mg CE/Kg FW [32]
Labiateae	*Ocimum basilicum*/ Basil		NA	TFC 12 mg QE/g DW [30]
	*Salvia officinalis*/ Sage	Flavones	Luteolin, 16.70 Apigenin, 1.20	
Chenopodiaceae	*Spinacia oleracea* /Spinach	Flavonols Flavones	Kaempferol, 6.38 Quercetin, 3.97 Myricetin, 0.35 Luteolin, 0.74	TFC 1800–3700 mg/Kg FW Kaempferol 9 mg/Kg FW [16,33]
	*Beta vulgaris*/ Chard	Flavonols	Kaempferol, 5.80 Myricetin, 3.10 Quercetin, 2.20	TFC 12 mg QE/g DW [30]
Convolvulaceae	*Ipomoea batatas*/ Purple sweet potato	Anthocyanins	Cyanidin, 10.60 Delphinidin, 0.90 Pelargonidin, 0.02	TFC 1.87–3.95 mg QE/g DW [34]
Solanaceae	*Solanum lycopersicum*/ Tomato	Flavanones Flavonols	Naringenin, 0.68 Quercetin, 0.58 Myricetin, 0.13 Kaempferol, 0.09	TFC 4–26 mg/100 g FW Kaempferol 8 mg/Kg FW [16,35]
	*Solanum tuberosum*/ Potato	Flavonols	Quercetin, 0.49	TFC 153 mg CE/Kg FW Kaempferol 46 mg/Kg FW Quercetin 4 mg/Kg FW Isorhamnetin 19 mg/Kg FW [16,18]
	*Caspicum annuum*/ Green sweet pepper	Flavonols Flavones	Quercetin, 2.21 Kaempferol, 0.06 Luteolin, 4.71	Luteolin 2.6 mg/Kg FW [16]
	*Caspicum frutescens*/ Green hot chilli pepper	Flavonols Flavones	Quercetin, 14.70 Myricetin, 1.20 Luteolin, 3.87 Apigenin, 1.40	TFC 27 mg CE/100 g FW Quercetin 2 mg/Kg FW Luteolin 25 mg/Kg FW Apigenin 5 mg/Kg FW [16,36]
	*Solanum melongena*/ Aubergine	Anthocyanins	Delphinidin, 85.69	Rutin 9 µg/mg DW (fruit skin) Delphinidin-3-rutinoside 1200 µg/mg DW (fruit skin) [37]
Cucurbitaceae	*Cucurbita pepo*/ Courgette	Flavonols	Quercetin, 0.66	Isorhamnetin 15 mg/Kg FW [16]
	*Cucurbita* spp./ Pumpkin	Flavonols Flavones	Luteolin, 1.63	TFC 20–40 mg QE/g FW Isorhamnetin 35 mg/Kg FW Kempferol 23 mg/Kg DW Luteolin 18 mg/Kg DW Apigenin 9 mg/Kg DW [16,38]
